# Neostigmine versus sugammadex on outpatient recovery among obese patients with obstructive sleep apnea: A randomized controlled trial

**DOI:** 10.1038/s41598-026-47043-2

**Published:** 2026-03-31

**Authors:** Rodney A. Gabriel, Brian P. Curran, Engy T. Said, Paige S. Tsuda, Dale N. Bongbong, Adam S. Deconde, Carol H. Yan, Jerry Ingrande

**Affiliations:** 1 Department of Anesthesiology, University of California, San Diego, 9400 Campus Point Dr. La Jolla, La Jolla, 92037 CA USA; 2 School of Medicine, University of California, San Diego, La Jolla, CA USA; 3 Department of Otolaryngology, University of California, San Diego, La Jolla, CA USA; 4 Department of Veteran Affairs Medical Center, La Jolla, CA USA

**Keywords:** Sugammadex, Neostigmine, Outpatient surgery, Obesity, Obstructive sleep apnea, Outcomes research, Clinical trial design

## Abstract

Patients presenting for outpatient surgery may have several comorbidities, including obesity and obstructive sleep apnea. The objective was to measure the efficacy of sugammadex (versus neostigmine) in reducing post-anesthesia care unit length of stay among obese patients with obstructive sleep apnea undergoing outpatient surgery. Furthermore, we aimed to assess differences in post- and pre-operative pulmonary function test and arterial blood gas metrics. We hypothesized that sugammadex would improve recovery times. This was a single institution randomized controlled double-masked clinical trial assessing the efficacy of sugammadex versus neostigmine in reducing length of stay among obese patients with obstructive sleep apnea undergoing outpatient surgery. Secondary outcomes included changes in pulmonary function test and arterial blood gas measurements postoperatively. Mann Whitney U test was the primary statistic used to compare primary and secondary outcomes. There were 90 subjects enrolled, divided equally into the neostigmine and sugammadex cohorts. The median [quartile] length of stay was 125 min [73, 186] versus 123 min [94, 169] in the neostigmine and sugammadex cohorts, respectively (*P* = 0.79). There were no statistically significant differences in proportional changes of pulmonary function tests (all *P* > 0.05) nor arterial blood gas measurements (all *P* > 0.05). This suggests that among patients with obesity and OSA undergoing low-to-moderate risk surgery in the outpatient setting, routine use of one reversal agent over the other would not affect overall PACU efficiency in an ambulatory surgery center. Trial registration: Clinicaltrials.gov NCT04570150, Principal investigator: Jerry Ingrande, Date of registration: 29/09/2020).

**Supplementary Information:**

The online version contains supplementary material available at 10.1038/s41598-026-47043-2.

## Introduction

Ambulatory surgery comprises a large proportion of annual surgical procedures^1^. Optimizing both patient safety and perioperative efficiency are key metrics for a successful outpatient surgery center^2,3^. Patients presenting for outpatient surgery may have several comorbidities^1^, including obesity and obstructive sleep apnea (OSA), and thus, it is important to appropriately optimize anesthetics to deliver safe and efficient perioperative care. Several outpatient surgeries require neuromuscular blockade for general endotracheal anesthesia. If the neuromuscular block is not appropriately reversed, it may lead to postoperative residual neuromuscular blockade, subsequent respiratory complications and prolonged recovery times^4^.

Two reversal agents used for neuromuscular blockade include sugammadex and neostigmine (in combination with glycopyrrolate); the former of which had been popularized around 2016 and its utilization since has been increasing in the United States^5^. Several randomized controlled trials have investigated the efficacy and superiority of sugammadex versus neostigmine^6–17^. Sugammadex has been demonstrated to be more efficacious in time to and/or degree of reversal of neuromuscular blockade in a variety of surgical populations^7,8,11,12,17,18^. Specifically, sugammadex decreased PACU length of stay following operative laryngoscopy^6^, bariatric surgery^9,18^, lung resection surgery^11^, and surgery to treat OSA^17^. A retrospective study demonstrated that sugammadex was not associated with improved perioperative times in obese patients undergoing bariatric surgery^19^. Thus, a key question remains regarding whether the use of sugammadex improves postoperative recovery in patients with both obesity and OSA undergoing outpatient surgery. Furthermore, there are limited data regarding reversal effects on pulmonary function tests^13^ and partial pressure of carbon dioxide (PaCO_2_) and oxygen (PaO_2_) in arterial blood. A previous clinical trial demonstrated that there was no difference in postoperative strength, as measured by spirometry, between those who received sugammadex versus neostigmine^13^. Thus, the objective of this single institution randomized controlled double-masked clinical trial was to measure the efficacy of sugammadex (versus neostigmine) in reducing PACU length of stay among obese patients with OSA undergoing outpatient surgery. Furthermore, we aimed to assess differences in post- and pre-operative pulmonary function tests (to assess postoperative strength) and arterial blood gas metrics. We hypothesized that sugammadex would improve PACU recovery times in this targeted patient population.

## Methods

This study was approved by the University’s Institutional Review Board (IRB #200866) and written informed consent was obtained from all subjects participating in the trial. The trial was registered prior to patient enrollment at clinicaltrials.gov (NCT04570150, Principal investigator: Jerry Ingrande, Date of registration: 29/09/2020). Subject enrollment began January 2021 and ended November 2022. This was a randomized controlled trial and no changes to methods were performed after trial commencement.

### Study participants

Written, informed consent was obtained from all participating subjects.

### Inclusion and exclusion criteria

This single institution study assessed the efficacy of sugammadex versus neostigmine for PACU recovery following general endotracheal tube anesthesia in obese patients with OSA following outpatient surgery. The inclusion criteria included patients with obesity (body mass index [BMI] ≥ 30 kg/m^2^), documented diagnosis of OSA with or without continuous positive airway pressure use (this was based on an active diagnosis of OSA in the participant’s medical record with confirmation of diagnosis within a clinical note from their primary care provider and/or pulmonologist within one year), and election of outpatient surgery requiring general anesthesia with neuromuscular blockade. Surgical categories (procedures in category) included were laparoscopic abdominal surgery (cholecystectomy, hernia repair), foot and ankle surgery (subtalar fusion), laparoscopic gynecologic surgery (hysterectomy, myomectomy), head and neck surgery (endoscopic sinus surgery, thyroidectomy, direct microlaryngoscopy) and plastic surgery (breast reconstruction, abdominoplasty). Exclusion criteria included patients with a history of hepatic, renal, cardiovascular and/or cerebrovascular dysfunction, history of difficult intubation, history of adverse reaction to anesthesia, and history of adverse reaction to rocuronium, sugammadex, and/or neostigmine/glycopyrrolate.

### Randomization of cohorts and perioperative management

Potential subjects were recruited on day of surgery. Patients were provided with informed consent. Those agreeable to participate were further randomized (via sequentially numbered opaque envelopes) to either the sugammadex cohort (S) or neostigmine cohort (N) in a 1:1 fashion. Both the subject and the investigator were masked to cohort assignment. The anesthesiologist providing anesthetic care to the subject was not masked. After consent was provided, baseline pulmonary function tests were captured using a MIR Spirobank USB Spirometer (MIR, Rome, Italy), to obtain forced expiratory volume in 1 s (FEV1), forced vital capacity (FVC) and peak expiratory flow rate (PEF) data. Baseline arterial blood gas was analyzed from arterial blood drawn (~ 1.5 cc) from the radial artery. The research coordinator or study investigator obtained the study drug from the research pharmacist, which was sealed in a non-transparent envelope to maintain blinding. This was provided to the anesthesiologist, who would then be informed of the study drug once envelope was opened. The study pharmacist prepared doses for sugammadex (2 mg/kg total body weight) and neostigmine (0.07 mg/kg total body weight; maximum dose 5 mg). We chose total body weight for dosing as this is not only recommended by the manufacturer, but is supported by pharmacokinetic studies specific to obese patients^20^. Furthermore, prior studies analyzing various weight scalars of sugammadex in obese patients demonstrated slower time to response when scalars other than total body weight were used^21^. Glycopyrrolate was dispensed in a dosage ratio of 0.2 mg glycopyrrolate to 1 mg neostigmine, with a maximum dose of 1 mg.

Intraoperatively, the patient underwent general endotracheal anesthesia with standard induction doses: propofol (1–2 mg/kg), rocuronium (0.6–1.2.6.2 mg/kg), and fentanyl (50-200mcg). Maintenance of anesthesia was at the discretion of the anesthesiologist and involved any combination of volatile anesthetic (sevoflurane), propofol, opioids, and muscle relaxation (rocuronium). The amount of rocuronium given perioperatively was not controlled and left to the discretion of the anesthesiologist. However, all subjects enrolled in this study experienced a complete neuromuscular block during their respective procedure. Standard pre-emptive anti-emetics were administered, including ondansetron and dexamethasone. Prior to administrating muscle relaxant reversal, authors ensured that there were a train-of-four of 4 twitches and a train-of-four ratio > 0.9 when monitored on the facial nerve (orbicularis oculi muscle).

In the PACU, spirometry (FEV1, FVC, and PEF) and arterial blood gas measurements (PaCO_2_ and PaO_2_) were assessed as a postoperative measurement approximately 30 min after patient arrival to PACU. Spirometry was administered by the anesthesiologist investigators (JI and RG). All spirometry measurements for both groups were performed with the patient in the sitting position. Supplemental oxygen was removed during the measurements.

### Outcome measurements

The primary outcome was PACU length of stay (minutes) defined as time from subject arrival to the PACU to time of discharge readiness (time at which subject is determined ready to be discharged from the PACU. This does not include time after patient is ready to be discharged but waiting for a ride). At our institution’s outpatient surgery center, discharge readiness was determined when the patient had a score of 8 or greater on the Modified Aldrete discharge scoring tool^22^. Patients with scores of zero in any category required evaluation by an anesthesiologist. In addition, patients need to have temperature greater that 96 degrees Fahrenheit, minimal nausea or vomiting, and pain adequately managed according to the patient’s acceptable level of pain. The secondary outcomes of interest include^1^: proportional changes of post versus pre-pulmonary function test measurements, including FEV1, FVC, and PEF; and^2^ proportional changes of post versus pre-arterial blood gas measurements, including PaCO_2_ and PaO_2_. No changes in the definition of the primary or secondary outcomes were done after trial commenced.

### Statistical analysis

We hypothesized that sugammadex compared to neostigmine would shorten PACU length of stay among obese patients with OSA following outpatient surgery at a freestanding academic surgery center. The primary outcome (PACU length of stay) and the secondary outcomes (proportional changes in pulmonary function tests and arterial blood gas) were continuous values. All data was assessed for normality using Shapiro-Wilk test prior to statistical analysis. Mann Whitney U test was used to compare outcomes between the S and N cohorts. Reported P-values were two-sided and was considered statistically significant if < 0.05. Next, we performed a multivariable linear regression to adjust for potential confounders for the log-normal of PACU length of stay. PACU length of stay followed a non-zero right-skewed distribution (Supplemental Fig. 1A), whereas the log-normal of PACU length of stay followed a normal distribution (Supplemental Fig. 1B). Use of log-normal conversions of length of stay has been used in previous operating room efficiency studies to convert the outcome into a normal distribution^23^. For this model, the dependent variable was the log-normal of PACU length of stay and the primary independent variable was experimental cohort (S versus N). Other independent variables included in the model were age (years), male sex, BMI (kg/m^2^), surgical case duration (minutes from patient arriving to exiting operating room), intraoperative opioid use (measured in mg of intravenous morphine equivalents [MEQ]), and surgery type. Reported from the regression model were the coefficient, 95% confidence interval (CI), P-value of each independent variable, and the R^2^ of the model to measure the proportion of variance in the dependent variable that is explained by the independent variables. All subjects were analyzed on an intention-to-treat basis. There was no missing data that required imputation. R V.4.2.2 (https://www. r- project. org/) was used for all analyses.

### Sample size justification

A total of 90 subjects (45 subjects in each group) were enrolled in this study. A total of 36 subjects in each group were needed to provide 80% power with an alpha of 0.05 to detect a 10-minute difference in time to PACU discharge, assuming a standard deviation of 15 min. In one article, the cost per minute in the PACU was calculated at approximately $12^24^. The difference in cost of 200 mg sugammadex versus neostigmine/glycopyrrolate can be estimated at about $100^25^. We chose 10 min because that would give a cost approximation for savings related to sugammadex if it reduced length of stay by this much time. Forty-one subjects in each group were necessary to ensure 80% power with an alpha of 0.05 to detect a 5 mmHg difference in PaCO_2_ (assuming a standard deviation of 8 mmHg) and a 25 mmHg difference in PaO_2_ (assuming a standard deviation of 40 mmHg). This sample size also allowed a detection of a 20% decrease in FVC from baseline.

## Results

**Fig. 1:**
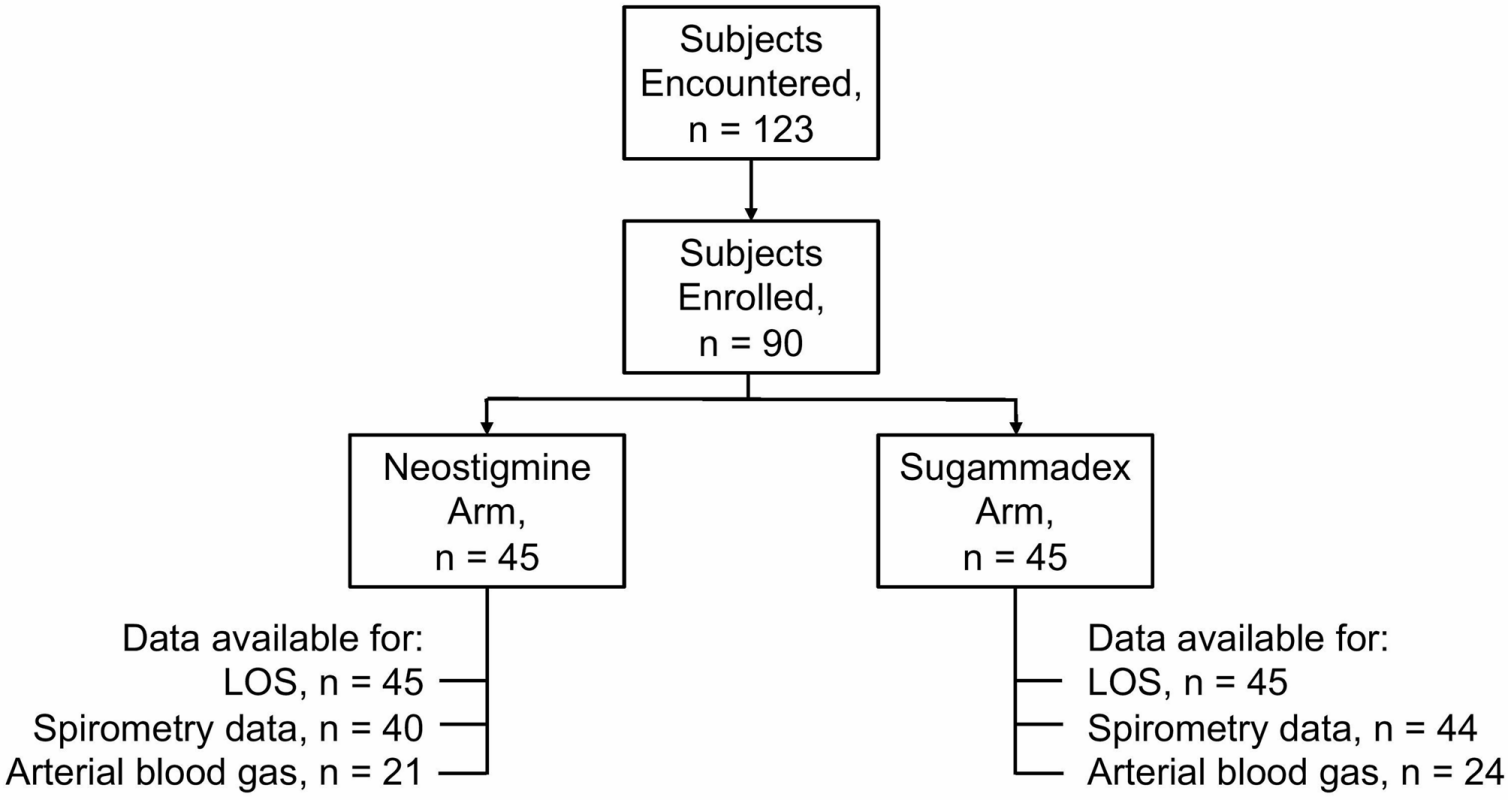
Consolidated Standards of Reporting Trials diagram of subject recruitment. Abbreviations: LOS, length of stay.

**Fig. 2:**
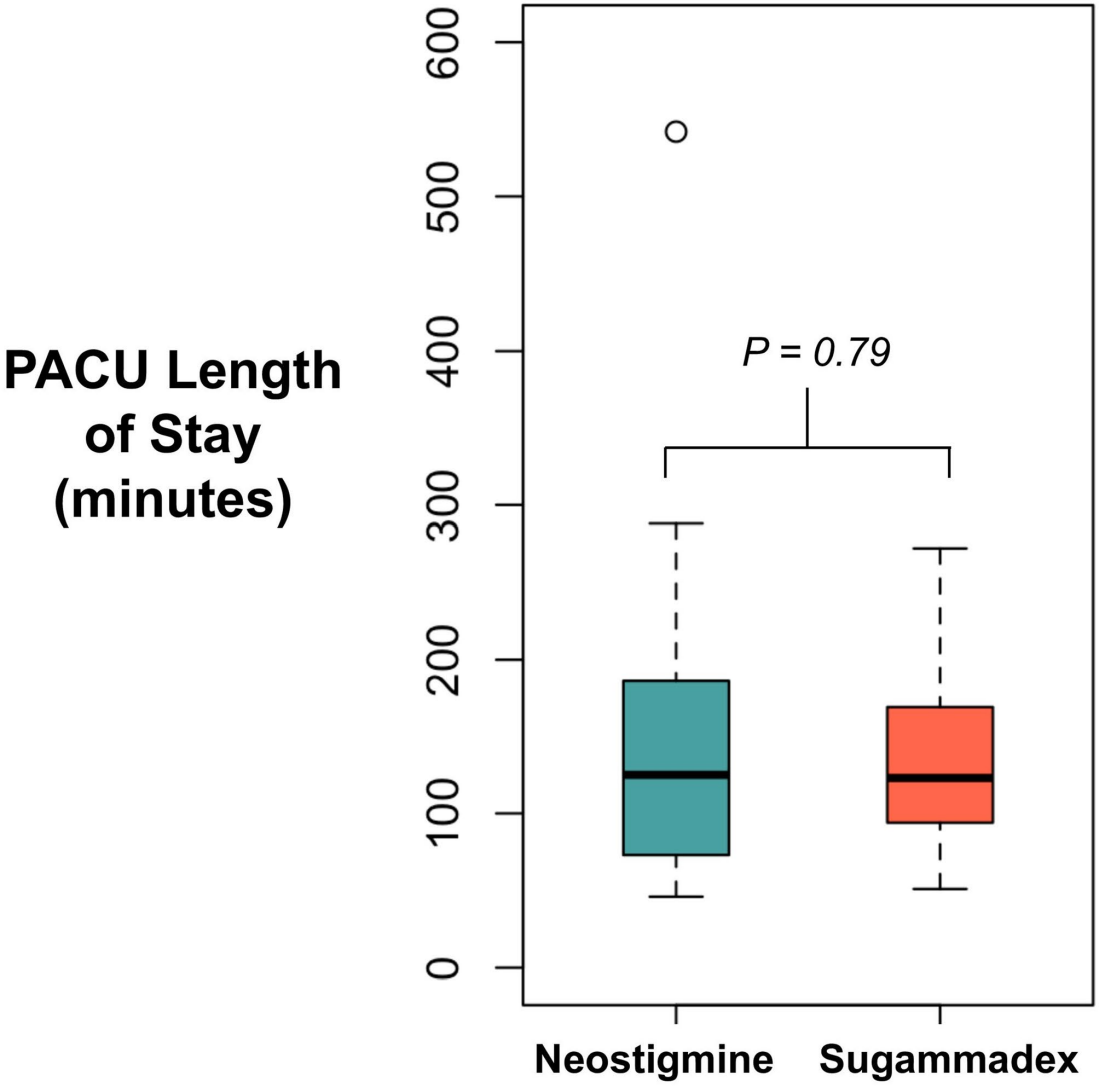
Box plot illustrating difference between sugammadex and neostigmine cohorts for PACU length of stay. Statistical differences were measured via Mann Whitney U test. Abbreviations: PACU, post-anesthesia care unit.

From January 2021 and ended November 2022, there were 90 subject enrolled, divided equally into the N and S cohorts (Fig. 1). Baseline characteristics, including age, sex, body mass index, case duration, intraoperative opioid use, and surgical procedure, were similar in both cohorts (Table 1). There was no difference in the median length of stay between cohorts (*P* = 0.79) (Fig. 2), in which the median [quartile] PACU length of stay was 125 min [73, 186] versus 123 min [94, 169] in the N and S cohorts, respectively. One subject (1.1%) in the N cohort had an immediate postoperative respiratory complication in the PACU due to inadequate neuromuscular blockade reversal, which was resolved after subsequent reversal with 4 mg/kg sugammadex. No subsequent complications were noted afterwards. There were no unplanned hospital admissions or adverse events in our outpatient surgical population.

**Table 1:** Baseline characteristics of the neostigmine and sugammadex cohorts. Wilcoxon rank sum test and Pearson’s Chi-squared test were used to compare differences in continuous and categorical variables, respectively.

	Neostigmine	Sugammadex	*P*-value
Total	45	45	
Age (years), median [quartiles]	49 [38, 60]	49 [38, 61]	0.86
Male sex, n (%)	15 (33.3)	13 (28.9)	0.82
Body mass index (kg/m2), median [quartiles]	35.2 [32.1, 37.8]	35.4 [33.0, 38.9]	0.32
Surgical case duration (minutes), median [quartiles]	126 [75, 180]	122 [92, 165]	0.96
Intraoperative opioid use (mg MEQ), median [quartiles]	20 [10, 20]	20 [10, 20]	0.49
Surgery			0.42
Laparoscopic abdominal surgery, n (%)	7 (15.6)	5 (11.1)	
Foot/Ankle surgery, n (%)	1 (2.2)	1 (2.2)	
Laparoscopic gynecological surgery, n (%)	8 (17.8)	4 (8.9)	
Head/Neck surgery, n (%)	29 (64.4)	33 (73.3)	
Plastics surgery, n (%)	0 (0)	2 (4.4)	

MEQ, intravenous morphine equivalents.

### Subgroup analysis of the primary outcome

Shapiro-Wilk test was used to assess for normality, which determined all outcomes followed a non-parametric distribution. We compared outcomes specifically for head/neck surgery and by quartiles of case duration. In the head and neck surgery cohort, there were 29 and 33 participants in the N and S cohorts, respectively. The median [quartiles] PACU length of stay were 125 [76, 186] minutes versus 115 [90, 159] minutes, respectively, with no statistically significant difference (*P*= 0.87). We binned subgroups for case duration based on the quartiles of case duration^1^:< 82.0 min^2^;≥ 82.0 and < 122.5 min^3^;≥ 122.5 min and < 175 min; and^4^≥ 175 min. When comparing the N versus S cohorts, for the first quartile subgroup, the median [quartiles] PACU length of stay were 56 [55, 69] minutes versus 68.5 [64.25, 80.25] minutes, respectively (*P* = 0.12). For the second quartile subgroup, the median [quartiles] PACU length of stay were 97.5 [92.75, 104.75] minutes versus 103.0 [98.25, 117.25] minutes, respectively (*P* = 0.21). For the third quartile subgroup, the median [quartiles] PACU length of stay were 145.0 [137.0, 155.5] minutes versus 145.0 [133.5, 156.5] minutes, respectively (*P* = 0.84). For the fourth quartile subgroup, the median [quartiles] PACU length of stay were 251.0 [214.0, 281.0] minutes versus 217.5 [197.75, 250.25] minutes, respectively (*P* = 0.34).

### Secondary outcomes

Pre- and post-operative spirometry data were available for 40 (88.9%) and 44 (97.8%) subjects in the N and S cohorts, respectively (Table 2). There was no difference in the proportional change of the post- versus pre-operative FEV1 measurements, in which the median [quartile] change was 0.73 [0.52, 0.87] and 0.63 [0.34, 0.88] in the N versus S cohorts, respectively (*P* = 0.22). There was no difference in the proportional change of the post- versus pre-operative PEF measurements, in which the median [quartile] change was 0.65 [0.47, 0.91] and 0.57 [0.32, 0.88] in the N versus S cohorts, respectively (*P* = 0.32). There was no difference in the proportional change of the post- versus pre-operative FVC measurements, in which the median [quartile] change was 0.72 [0.53, 0.94] and 0.62 [0.43, 0.93] in the N versus S cohorts, respectively (*P* = 0.31).

**Table 2:** Comparison of secondary outcomes in the neostigmine versus sugammadex cohorts. P-value was determined by the Mann Whitney U Test for comparing proprtional changes. Fisher exact test was used to compare differences in missing data between cohorts.

	Neostigmine	Sugammadex	*P*-value
Spirometry Results			
Total with available data	40	44	0.20
Proportional change in FEV1 (postop versus preop), median [quartiles]	0.73 [0.52, 0.87]	0.63 [0.34, 0.88]	0.22
Proportional change in PEF (postop versus preop), median [quartiles]	0.65 [0.47, 0.91]	0.57 [0.32, 0.88]	0.32
Proportional change in FVC (postop versus preop), median [quartiles]	0.72 [0.53, 0.94]	0.62 [0.43, 0.93]	0.31
Arterial Blood Gas Results			
Total with available data	21	24	0.67
Proportional change in PaO2 (postop versus preop), median [quartiles]	0.90 [0.66, 1.13]	0.74 [0.60, 0.91]	0.13
Proportional change in PaCO2 (postop versus preop), median [quartiles]	1.07 [0.95, 1.10]	1.08 [0.96, 1.18]	0.73

FEV1, forced expiratory volume in 1 s, FVC, forced vital capacity, PaO2, partial pressure of oxygen in the arterial blood, PaCO2, partial pressure of carbon dioxide in the arterial blood, PEF, peak expiratory flow rate.

Pre- and post-operative arterial blood gas data were available for 21 (46.7%) and 24 (53.3%) subjects in the N and S cohorts, respectively (Table 2). There was no difference in the proportional change of post- versus pre-PaCO_2_, in which the median [quartile] change was 1.07 [0.95, 1.10] and 1.08 [0.96, 1.18] in the N versus S cohorts, respectively (*P* = 0.73). There was no difference in the proportional change of post- versus pre-PaO_2_, in which the median [quartile] change was 0.90 [0.66, 1.13] and 0.74 [0.60, 0.91] in the N versus S cohorts, respectively (*P* = 0.13). Pre- and post-operative median values of results from arterial blood gases and pulmonary function tests are provided in Supplemental Table 1.

### Multivariable linear regression

Next, we modeled N versus S cohort to the log-normal of PACU length of stay while controlling for age, sex, BMI, case duration, intraoperative opioid use, and surgical procedure using multivariable linear regression. There was no difference in the N versus S cohorts in relation to the log-normal of PACU length of stay (coefficient = 0.09 [95% CI −0.02, 0.21], *P* = 0.11) (Table 3). Surgical case duration (*P* < 0.0001) was associated with the log-normal of PACU length of stay. The R^2^ of this multivariable regression model was 0.73.

**Table 3:** Results of multivariable linear regression modeling reversal agent to the log normal of post-anesthesia care unit length of stay (minutes) while controlling for age, sex, body mass index, surgical case duration, intraoperative opioid use and surgical procedure. The outcome was converted to log normal due to the non-normal distribution of this variable. The adjusted R-squared of the model was 0.731.

	Coefficient	*P*-value
Sugammadex versus neostigmine	0.09 [95% CI −0.02–0.21]	0.11
Age (decades)	0.02 [95% CI −0.02, 0.06)	0.32
Male sex (versus female sex)	−0.03 [95% CI −0.16, 0.10]	0.68
Body mass index (kg/m2)	−0.003 [95% CI −0.015, 0.009]	0.59
Surgical case duration (minutes)	0.005 [95% 0.004, 0.006]	< 0.001
Intraoperative opioid use (mg MEQ)	−0.002 [95% −0.01, 0.006]	0.65
Surgery		
Laparoscopic abdominal surgery	reference	
Foot/Ankle surgery	−0.09 [95% CI −0.50, 0.32]	0.68
Laparoscopic gynecological surgery	0.29 [95% CI 0.05, 0.53]	0.02
Head/Neck surgery	0.12 [95% −0.06, 0.30]	0.17
Plastics surgery	0.27 [95% CI −0.14, 0.68]	0.21

MEQ, intravenous morphine equivalents.

## Discussion

In this single institution randomized double-masked clinical trial, we demonstrated that there was no difference in PACU length of stay between sugammadex and neostigmine in obese patients with OSA undergoing outpatient surgery. Furthermore, there were no differences between cohorts in the changes from post- to pre-operative pulmonary function test and arterial blood gas measurements. This suggests that among patients with obesity and OSA undergoing low-to-moderate risk surgery in the outpatient setting, routine use of one reversal agent over the other would not affect overall PACU efficiency in an ambulatory surgery center. While some studies have demonstrated that incorporation of sugammadex into enhanced recovery after surgery pathways have been associated with reduced length of stay and cost^26^, our study demonstrates that this may not be the case for our particular study population.

Previous randomized controlled trials comparing sugammadex to neostigmine have targeted the obese patient population^18^. In a meta-analysis investigating its use in bariatric surgery, authors investigated the following outcome measurements: recovery time to train-of-four ≥ 0.9 (minutes), composite adverse events, residual neuromuscular blockade, and time to discharge from PACU^18^. Sugammadex was demonstrated to improve all outcomes. Specifically, the PACU time was reduced by approximately 27 min in this pooled analysis of bariatric surgery patients. In contrast, we found no difference in PACU length of stay among our study population which also targeted obese patients. However, there are several key differences between our study and theirs. Our study population included obesity ranges starting at ≥ 30 kg/m^2^ (rather than BMI ranges greater than 40 kg/m^2^). In addition, the surgeries included in both studies differed significantly. We focused on outpatient surgeries, which may have very different recovery profiles than patients undergoing bariatric surgery. In outpatient surgery, PACU recovery may already be streamlined at our institution with less incidence of delayed discharge times at baseline and decreased incidence of PACU complications. Obesity alone may not be an independent risk factor for prolonged PACU length of stay following outpatient surgery^27^. Furthermore, the surgical case durations in the bariatric surgery studies had shorter surgical case duration times compared to our study^18^. As we demonstrated a statistically significant correlation between surgical case duration and PACU length of stay, it is possible that the effect size between the two reversal agents were decreased given longer surgery times in our study compared to those included in that meta-analysis^18^. Thus, any improvement afforded by sugammadex may not be clinically apparent in our study population.

One previous study assessed the efficacy of sugammadex versus neostigmine in patients undergoing surgery for OSA^17^. This trial demonstrated that neuromuscular blockade reversal with sugammadex decreased incidence of postoperative complications and hospital costs when compared to reversal with neostigmine. They also demonstrated that operating room time and PACU length of stay was shortened by sugammadex. Several differences are notable between this and our study – namely, they excluded patients that were morbidly obese and that their specific surgical population was OSA surgery. Our study focused specifically on obese patients with a diagnosis of OSA undergoing outpatient surgery (not including surgery for OSA). The focus on airway surgeries may likely have a higher baseline of incidence of postoperative respiratory complications, unlike our outpatient surgical population, and, thus, any clinically significant differences may not be apparent from our study design.

Other retrospective observational studies had also demonstrated no benefits with sugammadex versus neostigmine in patients undergoing bariatric surgery^19^ and laparoscopic cholecystectomy^28^. In a retrospective analysis of patients undergoing bariatric surgery, sugammadex was not associated with reduced PACU length of stay. Their study population may be similar to ours given that this was an obese population without moderate to severe comorbidity burden and, thus, supports our findings^19^.

Several studies have demonstrated superior efficacy of sugammadex compared to neostigmine in relation to time to and degree of neuromuscular blockade reversal and incidence of postoperative respiratory complications^7,8,11,12,17,18^. Furthermore, in a large multicenter retrospective analysis of twelve institutions consisting of ~ 50,000 patients, sugammadex was associated with lower incidence of major pulmonary complications among a generalizable cohort of adult patients^29^. Despite the notable benefits of improved neuromuscular blockade reversal, we did not see any clinically significant benefits in terms of PACU length of stay in our targeted population. This likely may be due to the already low incidence of postoperative complications in the outpatient surgical population despite having obesity of OSA. Furthermore, we found no differences in pulmonary function tests and arterial blood gas measurements in both cohorts. Perhaps with a higher sample size for this specific surgical population, clinically significant benefits may be observed regarding postoperative respiratory complications, which also would translate to shortened PACU length of stay. Thus, it is unclear if routine use of sugammadex in obese and OSA patients undergoing outpatient surgery is beneficial, especially due to the cost differences between the two reversal agents. However, several studies have reported the cost-effectiveness of sugammadex versus neostigmine^17,30,31^. In a meta-analysis, sugammadex was associated with reduce postoperative respiratory complications in obese patients^29^. In the United States, the average cost per vial for sugammadex can be approximately $148 and $271 for the 200 mg and 500 mg doses, respectively, while neostigmine and glycopyrrolate can be estimated at $7-$34 and $3-$18, respectively^25^. Despite the increased drug cost, it appeared the cost savings associated with sugammadex may be partially due to the reduced costs associated with the decreased postoperative pulmonary complications with this drug^29,32^. Thus, when accounting for cost-benefit logistics, use of sugammadex versus neostigmine/glycopyrrolate should consider cost savings related to reductions in complications and length of stay, which would be dependent on the patient mix and volume.

One patient in the neostigmine group experienced a post-operative respiratory complication requiring sugammadex for rescue. Despite the fact that all patients were deemed to be fully recovered from neuromuscular blockade based upon train-of-four ratio, the risk of post-operative residual neuromuscular blockade is not obviated. Furthermore, while ABG and spirometric measurements may appear to be identical between groups, clinical safety may favor the use of sugammadex, especially in at risk-populations (i.e., obese individuals with OSA).

There are several limitations related to this study related to unavoidable constraints. We were likely under-powered to identify differences in changes for pulmonary function tests and arterial blood gas results as the study was not adequately powered for the secondary outcomes given the presence of missing arterial blood gas data. Arterial blood gas data was not available for a significant portion of the study population due to non-random events related to patient refusal for repeat needle stick in the PACU. Furthermore, the duration of PACU length of stay is dependent of several factors, not just neuromuscular blockade completeness, and thus, finding clinically significant differences in the metric among outpatient surgery may be difficult to ascertain with the current study design, especially given the low incidence of postoperative pulmonary complications in the PACU. Finally, missing spirometry data amongst the groups was due to patient refusal during the PACU period. We did not perform imputation, therefore spirometric outcomes should be interpreted cautiously.

Furthermore, our study design had notable methodological weaknesses. First, it was a single institution study focused on outpatient surgery at a freestanding surgery center. For more definitive results, it would be ideal to have representation of outpatient surgeries from more than one institution. Second, we dosed sugammadex based on total body weight rather than ideal body weight, which could theoretically influence outcomes. However, we describe in our methods the reasons for using the former^20,21^. Third, the surgical population was not homogeneous and included five different surgical specialties. However, our study was designed to analyze obese patients with concomitant diagnosis of OSA requiring general anesthesia with muscle relaxation undergoing outpatient surgery in our ambulatory surgical center. With an anticipated sample size of 90 subjects and strict inclusion criteria, it was not feasible to constrain our potential subject pool based upon surgical procedure. However, despite the heterogeneity of surgical procedures, our study population, anesthetics, and duration of anesthesia exposure were similar amongst subjects. Hence, differences in response to the anesthetic, and pharmacodynamic variability regarding response to neuromuscular blockade and reversal is likely to be small. Subgroup analyses were post-hoc and exploratory; results are therefore presented descriptively, without multiplicity correction, and should be interpreted with caution. Fourth, in regard to spirometry measurements, spirometry was not formally quality-graded according to American Thoracic Society/European Respiratory Society reproducibility standards, and therefore measurement variability cannot be excluded. Of note, a 10-minute effect size difference was chosen to determine sample size as prior studies have reported an accelerated PACU discharge of similar magnitude^33^.

Furthermore, we excluded patients with moderate to severe comorbidities, which could limit the generalizability to higher risk patients. However, our study targeted a population more appropriate for a freestanding ambulatory surgery. Higher risk patients generally do not qualify for outpatient surgery and are scheduled for surgery in our main hospital. There remains controversy whether obese patients with OSA are appropriate for outpatient surgery. Our study was specifically designed to analyze the effect of sugammadex versus neostigmine on obese patients with OSA undergoing outpatient surgery. Had our study found an effect, recommendations could have been made regarding appropriate reversal strategies. We routinely reversed our patients when the train-of-four was > 0.9 based on the orbicularis oculi monitoring (which may be more sensitive than adductor pollicis), thus this technique may explain limited difference between groups (due to a potential ceiling effect).

## Conclusions

Sugammadex did not decrease PACU length of stay or improve pulmonary function test or arterial blood gas changes in patients with OSA and obesity undergoing outpatient surgery. It is unclear whether routine use of sugammadex versus neostigmine is warranted in this specific surgical population; however, previous studies would suggest cost-savings with sugammadex likely related to its association with reduced postoperative pulmonary complications in the general population.

## Supplementary Information

Below is the link to the electronic supplementary material.

Supplementary material 1 (DOCX 32.2 kb)

Supplementary material 2 (JPG 746.7 kb)

Supplementary material 3 (DOCX 15.3 kb)

## Data Availability

Data will be made available upon reasonable request and with proper data use agreements.

## Abbreviations

BMI: Body mass index
FEV1: Forced expiratory volume in 1 s
FVC: Forced vital capacity
N: Neostigmine cohort
OSA: Obstructive sleep apnea
PACU: post-anesthesia care unit
PEF: Peak expiratory flow rate
S: Sugammadex cohort
