# Supplementary figures and images for: Neostigmine versus sugammadex on outpatient recovery among obese patients with obstructive sleep apnea: A randomized controlled trial
Source: Sci Rep. 2026 Mar 31;16:15567. doi: 10.1038/s41598-026-47043-2 (PMC13187052; doi:10.1038/s41598-026-47043-2)

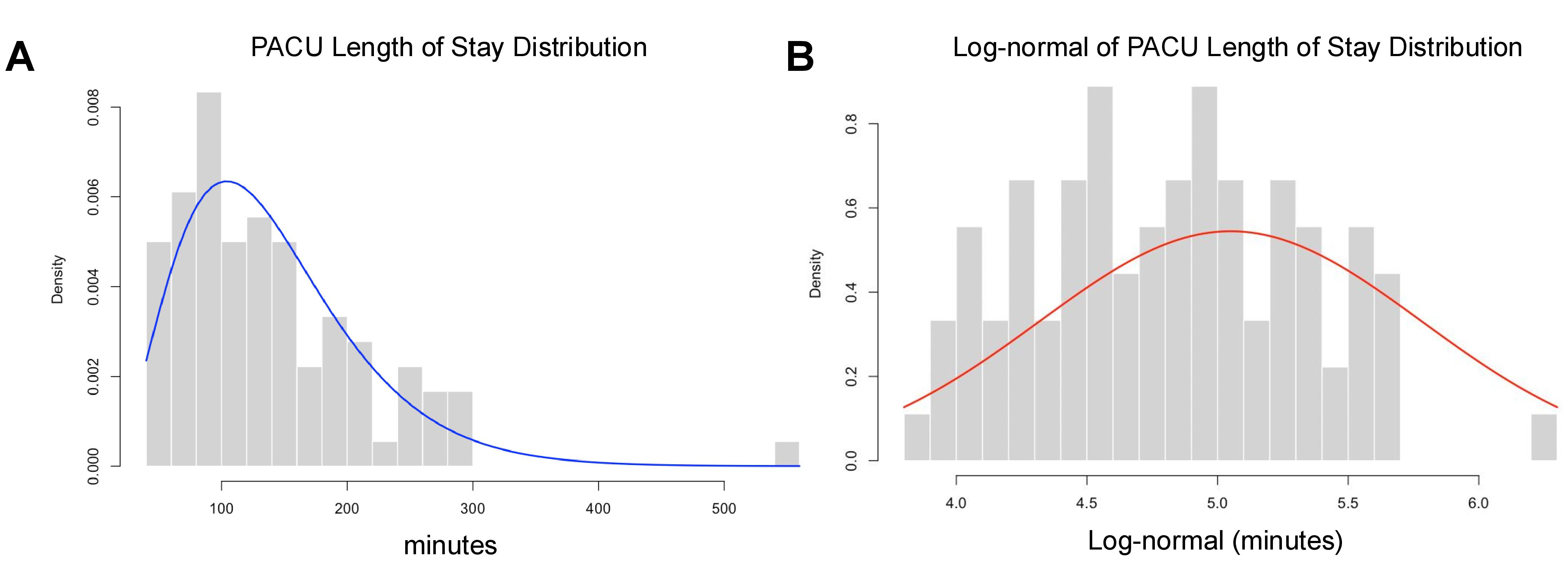

Supplement: Supplementary file 2 — Supplementary material 2 (JPG 746.7 kb) [file 41598_2026_47043_MOESM2_ESM.jpg]
