# Supplementary material for: Neostigmine versus sugammadex on outpatient recovery among obese patients with obstructive sleep apnea: A randomized controlled trial
Source: Sci Rep. 2026 Mar 31;16:15567. doi: 10.1038/s41598-026-47043-2 (PMC13187052; doi:10.1038/s41598-026-47043-2)
Supplement: Supplementary file 3 — Supplementary material 3 (DOCX 15.3 kb) [file 41598_2026_47043_MOESM3_ESM.docx]

|  | **Neostigmine cohort** | **Sugammadex cohort** | **P-value** |
| --- | --- | --- | --- |
| **Arterial Blood Gas** |  |  |  |
| Preop PaO2, median [quartiles] | 83.0 [71.0, 97.0] | 84.0 [79.75, 93.25] | 0.19 |
| Postop PaO2, median [quartiles] | 68.5 [58.0, 94.75] | 67.0 [59.0, 78.25] | 0.52 |
| Preop PaCO2, median [quartiles] | 40.3 [39.7, 41.3] | 41.25 [39.0, 42.075] | 0.63 |
| Postop PaCO2, median [quartiles] | 43.05 [40.025, 45.6] | 45.1 [43.575, 46.3] | 0.21 |
|  |  |  |  |
| **Pulmonary Function Tests** |  |  |  |
| Preop FEV1, median [quartiles] | 2.285 [1.698, 2.81] | 2.115 [1.785, 2.688] | 0.78 |
| Postop FEV1, median [quartiles] | 1.680 [1.180, 2.205] | 1.42 [0.91, 2.20] | 0.49 |
| Preop PEF, median [quartiles] | 3.745 [2.238, 4.740] | 2.925 [2.365, 4.590] | 0.88 |
| Postop PEF, median [quartiles] | 2.310 [1.445, 3.505] | 2.320 [1.173, 3.370] | 0.76 |
| Preop FVC, median [quartiles] | 2.715 [2.300, 3.655] | 2.605 [2.140, 3.105] | 0.39 |
| Postop FVC, median [quartiles] | 2.160 [1.555, 3.135] | 1.925 [1.310, 2.692] | 0.41 |
| **Supplemental Table 1**. Median and quartiles of pre and post arterial blood gas and pulmonary function test values between the neostigmine and sugammadex cohorts | | | |
| Abbreviations: |  |  |  |
| FEV1, forced expiratory volume in 1 second | |  |  |
| FVC, forced vital capacity |  |  |  |
| PaO2, partial pressure of oxygen in the arterial blood | |  |  |
| PaCO2, partial pressure of carbon dioxide in the arterial blood | | |  |
| PEF, peak expiratory flow rate |  |  |  |
